# Pregnancy outcomes after assisted reproductive procedures with embryos that had been derived from affected and unaffected ovaries among women with small unilateral endometriomas

**DOI:** 10.1002/rmb2.12020

**Published:** 2017-03-22

**Authors:** Akiko Takashima, Naoki Takeshita, Toshihiko Kinoshita

**Affiliations:** ^1^ Department of Obstetrics and Gynecology Toho University Medical Center Sakura Hospital Sakura Japan

**Keywords:** assisted reproductive technology, endometrioma, female infertility, in vitro fertilization, ovarian reserve

## Abstract

**Aim:**

To clarify the effects of small endometriomas on in vitro fertilization (IVF) outcomes. In the present study, the potential impact of small ovarian endometriomas on the quantitative and qualitative outcomes of IVF was evaluated in the same individual.

**Methods:**

A retrospective analysis was performed, in which 118 infertile women with unilateral endometriomas that were <40 mm in size and who underwent IVF or intracytoplasmic sperm injection were evaluated. Single frozen embryo transfer cycles were performed, with separate data collections for both the affected and the unaffected ovaries, which allowed for an evaluation of the implantation rate.

**Results:**

The mean antral follicular count and the number of follicular flushings, retrieved oocytes, and obtained embryos were significantly lower for the endometrioma‐containing ovary than for the contralateral, intact ovary. No significant difference was observed regarding the blastocyst retrieval and good‐quality blastocyst retrieval rates, pregnancy rate, and clinical pregnancy or live birth rate.

**Conclusion:**

Although the patients with a small endometrioma had a decreased ovarian reserve, they had lower pregnancy rates. The decision to transfer an embryo from an endometrioma‐containing ovary or from a contralateral, intact ovary also might not influence the pregnancy rate.

## 1 Introduction

Endometriosis is a disorder that is characterized by the presence and growth of endometrial tissue in ectopic sites.^1^ Although the reported prevalence among asymptomatic women ranges from 2% to 20%, the prevalence in women with dysmenorrhea is as high as 40%‐60%.^2^ Endometriosis also is frequently associated with infertility and affects >30% of infertile women.^3^ Increasing numbers of women with endometriosis have achieved pregnancy with assisted reproductive technology (ART). Several studies have suggested that the presence of an endometrioma reduces the quality of the oocytes, as reflected by the reduced rate of fertilization and implantation after controlled ovarian hyperstimulation and in vitro fertilization (IVF) treatment.^4^, ^5^, ^6^, ^7^ The European Society of Human Reproduction and Embryology guidelines state that there is no evidence that cystectomy prior to treatment with ART improves the pregnancy rates in infertile women with endometriomas that are >30 mm. The Guideline Development Group recommends that clinicians only consider cystectomy prior to ART to improve endometriosis‐associated pain or the accessibility of the follicles.^8^ In addition, it has been reported that surgery prior to scheduled ART does not benefit asymptomatic women with an endometrioma.^9^

However, no study has evaluated the impact of small endometriomas and the ideal treatment schedule for IVF/intracytoplasmic sperm injection (ICSI) has yet to be defined. The quantitative ovarian reserve, embryo quality, and IVF outcome of women with a small endometrioma who undergo ART but with a high average age remain a matter of debate because relevant evidence is scarce. Therefore, further studies are needed to determine whether the endometrioma plays a role in the decrease of fecundity or in the lower pregnancy rates after IVF. Thus, the objective of the present study was to evaluate the quantitative and qualitative IVF outcomes in ovaries with and without a small (especially <40 mm) endometrioma in the same individual, with a high average age of the participants.

## 2 Materials and Methods

### 2.1 Patients

In this retrospective analysis, 118 infertile women with a unilateral endometrioma that was <40 mm and who had undergone IVF or ICSI from April 2011 to March 2014 were examined. All the patients met the following inclusion criteria: (1) no previous history of adnexal surgery; (2) both ovaries were present; (3) a diagnosis of a unilateral endometrioma; (4) a menstrual cycle duration that ranged from 25 to 35 days; (5) no clinical sign of hyperandrogenism; and (6) a Body mass index (BMI) that ranged from 18 to 25 kg/m^2^. The exclusion criteria were: (1) a previous history of any systematic disease or malignancy; and (2) older than 45 years.

An ultrasound‐based diagnosis of endometrioma was achieved following the visualization of persistent, round, homogeneous hypoechoic “tissue” with low‐level echoes within the ovary, as previously described.^10^ Each endometrioma was measured in three dimensions and the mean diameter was calculated. The contralateral, intact ovary was confirmed to not contain an endometrioma via transvaginal ultrasound.

Written informed consent was obtained from all the patients. This study was approved by the Ethical Review Board of Toho University Medical Center Sakura Hospital, Sakura, Japan.

### 2.2 Measurement of the serum hormone levels

Samples of blood (5 mL) were collected on day 3 of menstruation during the IVF cycle and were centrifuged. The resultant serum samples were frozen at −80°C until subsequent assessments of the levels of anti‐Müllerian hormone (AMH), follicle‐stimulating hormone (FSH), and estradiol (E2) were performed. The serum AMH levels were measured with a commercial assay kit (AMH Gen II ELISA; Beckman Coulter, Brea, CA, USA). The detection limit of this kit was 0.16 ng/mL.

### 2.3 Cycle monitoring during the infertility treatment

The number of early antral follicles was determined by ultrasound examination on day 3 of IVF. Ultrasonographic (USG) measurements were performed with a multifrequency transvaginal probe (Voluson P6 with a multifrequency convex endovaginal transducer; GE Healthcare Company, Tokyo, Japan), as previously described.^11^ Briefly, on day 3, all the antral follicles with a mean diameter of 2‐10 mm were counted in both the endometrioma‐containing ovary and in the contralateral, intact ovary. All the participants were treated according to a standard treatment protocol for FSH‐mediated ovarian hyperstimulation, using a short‐acting antagonist to gonadotropin‐releasing hormone, with 10 000 units of human chorionic gonadotropin (hCG), which was used to induce follicular maturation at 36 hours before the collection of the eggs. The IVF or ICSI procedure was performed at 4–6 hours after collection of the ova. For this study, the oocytes were routinely stored separately according to the ovary of origin. All the embryos that reached an appropriate blastocyst stage were cryopreserved on day 5 or 6. The patients who prepared for frozen transfer cycles underwent programmed hormone replacement. Single frozen embryo transfer cycles were performed for 81 blastocysts, which were scored according to the Gardner standard.^12^ The blastocyst quality was classified as “good” (≥3 BB) or “poor” according to its trophectoderm and inner‐cell mass quality scores. The decision to transfer a given embryo was based on morphology, which indicated the best embryo. The ovary of origin did not influence the clinical choice. The appropriate FSH and human menopausal gonadotropin (hMG) dose was determined on an individual basis according to the antral follicular count (AFC) and the observation of the growing follicles. The number and size of the follicles that responded to FSH/hMG stimulation were monitored regularly by transvaginal sonography.

The ovarian response was assessed by the total dose of gonadotropin (recombinant FSH or hMG) that was required for ovarian stimulation, the number of follicles on the day that the oocyte was obtained, the number of retrieved oocytes, and the numbers of embryos that were obtained from the endometrial ovary and the contralateral, intact ovary.

“Pregnancy” was defined as a positive serum β‐hCG test and “clinical pregnancy” was defined as the ultrasonographic demonstration of an intrauterine gestational sac 4 weeks after the embryo transfer.

### 2.4 Clinical characteristics and outcome measures

The patients’ age, BMI, duration of the menstrual cycle, and tumor size were recorded as the baseline clinical characteristics. In addition, on day 3, the serum levels of the AMH, FSH, and E2 were recorded, along with the total number of doses and the duration of recombinant FSH/hMG stimulation. The following were evaluated and compared for the endometrioma‐containing ovary and the contralateral, intact ovary: the AFC, the degree of follicular flushing (follicular size: ≥17 mm), the oocytes that were retrieved, the embryos that were obtained, the blastocyst retrieval rate, the good‐quality blastocyst retrieval rate, the pregnancy rate, the clinical pregnancy rate, and the live birth rate. The primary outcome was the clinical pregnancy rate per cycle. The ovarian response to gonadotrophin stimulation was considered as a secondary outcome.

### 2.5 Statistical analysis

The chi‐square test or Fisher's exact test was used to compare the categorical variables. In order to compare the means of the continuous variables, the non‐parametric Mann‐Whitney *U* test was used if the continuous variables were not normally distributed and the Student's *t* test was used if the continuous variables were normally distributed. *P*‐values of <.05 were considered to be significant. The results were expressed as the arithmetic mean±SD, range, and median. JMP software (SAS Institute, Inc., Cary, NC, USA) was used for the statistical analysis.

## 3 Results

In total, 118 women were evaluated in this study and their clinical characteristics, as well as those of their endometriotic tumor, are summarized in Table 1. Their mean age was 40.4±0.9 years. Table 2 presents the comparisons of the IVF/ICSI outcomes for the ovaries with and without an endometrioma. The mean AFC, the number of follicular flushings, the retrieved oocytes, and the number of obtained embryos were significantly lower for the endometrioma‐containing ovaries than for the contralateral, intact ovaries. No significant difference was found with respect to the rate of blastocyst retrieval and the rate of good‐quality blastocyst retrieval. The total pregnancy rate, the clinical pregnancy rate, and the live birth rate were not significantly different (Table 3). When the study group was divided according to its pregnancy status (pregnant vs non‐pregnant), no significant difference was observed in the AMH level (1.72 [1.45‐1.99] vs 2.2 [<0.16‐4.7] ng/mL).

**Table 1:** Baseline clinical characteristics of the patients

Characteristic	Mean±SD
Age (years)	40.40±.90
Body Mass Index (kg/m^2^)	22.40±2.50
Menstrual cycle (day)	29.90±1.20
Duration of infertility (years)	3.20±.80
Size of the cyst (mm)	30.30±1.40
AMH (ng/mL)	1.75±.30
Day3 FSH (mIL/mL)	5.80±.60
Day3 E2 (pg/mL)	61.90±5.40
Total dose of FSH/hMG (IU)	2406.20±370.80
IVF/ICSI (%)	
IVF	38.10
ICSI	61.90

AMH, anti‐Müllerian hormone; E2, estradiol; FSH, follicle‐stimulating hormone; hMG, human menopausal gonadotropin; ICSI; intracytoplasmic sperm injection; IVF, in vitro fertilization.

**Table 2:** Comparing the in vitro fertilization/intracytoplasmic sperm injection outcome in the endometrioma and in the contralateral, intact ovary (mean±SD)

Variable	Endometrioma	Non‐endometrioma	*P*‐value
Antral follicle count	2.2±0.5	4.0±0.4	<.05
Antral follicle count	3.3±0.3	4.8±0.7	<.05
Number of oocytes	2.1±0.3	3.9±0.3	<.05
Number of obtained embryos	1.2±0.2	2.4±0.2	<.05
Blastocyst embryos (%)	37.1	31.5	NS
Good‐quality blastocyst embryos (%)	12.3	10.8	NS

NS, not significant.

**Table 3:** Comparing the frozen embyo transfer outcome in the endometrioma and in the contralateral, intact ovary

Variable	Endometrioma	Non‐endometrioma	*P*‐value
	N (%)	N (%)	
Transfer of a good‐quality embryo			
Pregnancy/embryo transfer (ET)	5/14 (35.7)	6/20 (30.0)	NS
Clinical pregnancy/ET	4/14 (28.6)	5/20 (25.0)	NS
Live birth/ET	3/14 (21.4)	4/20 (20.0)	NS
Transfer of a poor‐quality embryo			
Pregnancy/ET	2/19 (10.5)	4/28 (14.3)	NS
Clinical pregnancy/ET	1/19 (5.3)	3/28 (10.7)	NS
Live birth/ET	1/19 (5.3)	2/28 (7.1)	NS

NS, not significant.

In this study, no complication (eg, the spread of infection) was observed. In some cases, contamination of the follicular fluid was difficult to avoid during the retrieval of the oocytes in the endometrioma‐containing ovary. In all, seven of the 118 endometrioma‐containing ovaries were situated in a deep position relative to the surface; however, these ovaries were able to be reached and the follicles were able to be punctured in all cases.

## 4 Discussion

The objective of the present study was to evaluate the quantitative and qualitative IVF outcomes of women with and without a small endometrioma. Overall, the results suggested that the presence of an ovarian small endometrioma reduces the ovarian reserve but does not significantly affect the pregnancy rate.

The findings of this study were unique because the single embryo transfer policy that was followed, together with the separate data collections for both the affected and the unaffected ovaries, allowed for the evaluation of the implantation rate. The implantation rate thus could be directly attributed to the ovary with the endometrioma or to the contralateral, healthy ovary. Embryo cryopreservation is a routine part of ART that is associated with many benefits. Most studies have suggested that cryopreservation maximizes the cumulative success of each and every IVF cycle.

The management of endometriomas that are <40 mm in size in women who attempt to conceive remains a matter of debate.^5^, ^13^, ^14^, ^15^ The results of this study demonstrated that the AFC, the number of follicular flushings, the number of retrieved oocytes, and the number of obtained embryos were all significantly lower for the endometrioma‐containing ovary than for the contralateral, intact ovary. These results were limited only to cysts that are <40 mm in size, which implies that small endometriomas can quantitatively decrease the ovarian reserve.

Few studies have compared the numbers of oocytes that are produced by endometrioma‐containing ovaries and healthy ovaries in the same individual. One of the first to compare the responses of affected and unaffected ovaries to gonadotropins showed the production of a reduced number of co‐dominant follicles in endometrioma‐containing ovaries in response to ovarian stimulation and this reduction was more evident in women with endometriomas that were >20 mm in size.^5^ Another study also reported that the presence of an endometrioma resulted in a reduced response to ovarian stimulation, compared with the response of the contralateral, intact ovary in the same individual. It also was found that, in the case of endometriomas that are <30 mm, the basal FSH concentration is the most important prognostic factor for oocyte retrieval.^14^ In contrast, it has been reported that the presence of an endometrioma is not associated with a reduction in the number of retrieved oocytes.^16^ In the present study, no statistically significant difference was observed in the percentage of good‐quality blastocysts that was able to be retrieved, the pregnancy rate, the clinical pregnancy rate, and the live birth rate between the ovaries. Therefore, it is believed that small endometriomas might not be involved in a decrease in oocyte quality and might not affect pregnancy outcomes. The decision to transfer an embryo from an endometrioma‐containing ovary or from a contralateral, intact ovary also might not influence the pregnancy rate.

Laparoscopic ovarian surgery is a major topic in the field of reproductive medicine and the excision of ovarian endometriomas has been reported to be associated with detrimental effects on the ovarian reserve immediately after surgery.^17^ In addition, previous studies have demonstrated that both the number of follicles and the number of oocytes that are retrieved during ovarian hyperstimulation are markedly reduced in the surgically resected gonad, compared with the contralateral, intact ovary.^18^, ^19^ Thus, it appears that the surgical removal of endometriomas could exert a negative effect on fertility.

The precise risks that are associated with oocyte retrieval following treatment for an endometrioma are not well known. According to one study, at least five complications can be associated with a conservative approach in patients who are undergoing IVF cycles: (1) rupture of the endometrioma; (2) development of a pelvic abscess; (3) a failure to recognize an occult, early‐stage malignancy; (4) difficulties in oocyte retrieval; (5) contamination of the follicular fluid with the contents of the endometrioma; and (6) the progression of endometriosis.^18^ All of these risk factors should be considered when the method of oocyte retrieval is determined. In this study, no complication was observed. The results of this study demonstrated that lower reserves were not associated with a lower pregnancy rate. Therefore, this finding implies that the appropriate choice of management of a small endometrioma for a woman with a high average age should be the avoidance of surgical intervention.

In this study, no significant difference was observed in the AMH levels between the pregnant and the non‐pregnant women. Although some studies have reported that AMH can be a predictor of the ovarian reserve and the success rate of IVF,^20^, ^21^ this study's findings are consistent with those of other studies.^22^, ^23^

Several limitations affect the results of the present study. First, this study included only a small number of participants with a relatively high average age (40.4±0.9 years). The overall pregnancy rate in this study was relatively low but could be explained by the high average age of the participants, as the overall pregnancy rate was age‐appropriate. The findings of this study were unique, in that the average age was relatively higher than that in similar studies. Other studies also were limited to patients who were younger than 40 years of age at the time of the IVF/ICSI cycle.^5^, ^14^ The patients who receive IVF/ICSI in Japan tend to be older. Most of the patients were older than 40 years of age in this study. Second, transvaginal USG‐based diagnosis without a laparoscopy was performed in this study. A staging system for endometriosis, based on the revised American Society for Reproductive Medicine score classification and the Endometriosis Fertility Index for an accurate diagnosis, has not yet been developed.

In conclusion, despite the small number of participants in the present study, it was concluded that women with a small endometrioma and with an average age of 40 years have a reduced ovarian reserve but this does not significantly affect the pregnancy rate. A large, prospective study therefore is necessary in order to clarify the effects of small endometriomas on IVF outcomes.

## Disclosures

*Conflict of interest*: The authors declare no conflict of interest. *Human rights*: All the procedures that were followed were in accordance with the ethical standards of the responsible committees on human experimentation (institutional and national) and with the Helsinki Declaration of 1964 and its later amendments. Informed consent was obtained from all the patients to be included in the study. *Animal rights*: This article does not contain any study with animal participants that was performed by any of the authors.

## References

[1] Giudice LC , Kao LC . Endometriosis. Lancet. 2004;364:1789–1799. 15541453 10.1016/S0140-6736(04)17403-5

[2] Kyama CM , Debrock S , Mwenda JM , D'Hooghe TM . Potential involvement of the immune system in the development of endometriosis. Reprod Biol Endocrinol. 2003;1:1–19. 10.1186/1477-7827-1-123 PMC305339 14651748

[3] Augoulea A , Alexandrou A , Creatsa M , Vrachnis N , Lambrinoudaki I . Pathogenesis of endometriosis: the role of genetics, inflammation and oxidative stress. Arch Gynecol Obstet. 2012;286:99–103. 22546953 10.1007/s00404-012-2357-8

[4] Gupta S , Agarwal A , Agarwal R , Loret de Mola JR . Impact of ovarian endometrioma on assisted reproduction outcomes. Reprod Biomed Online. 2006;13:349–360. 16984764 10.1016/s1472-6483(10)61439-3

[5] Somigliana E , Infantino M , Benedetti F , Arnoldi M , Calanna G , Ragni G . The presence of ovarian endometriomas is associated with a reduced responsiveness to gonadotropins. Fertil Steril. 2006;86:192–196. 16716316 10.1016/j.fertnstert.2005.12.034

[6] Coccia ME , Rizzello F , Mariani G , Bulletti C , Palagiano A , Scarselli G . Impact of endometriosis on in vitro fertilization and embryo transfer cycles in young women: a stage‐dependent interference. Acta Obstet Gynecol Scand. 2011;90:1232–1238. 21793811 10.1111/j.1600-0412.2011.01247.x

[7] Harb HM , Gallos ID , Chu J , Harb M , Coomarasamy A . The effect of endometriosis on in vitro fertilization outcome: a systematic review and meta‐analysis. BJOG. 2013;120:1308–1320. 23834505 10.1111/1471-0528.12366

[8] Dunselman GA , Vermeulen N , Becker C , et al. ESHRE guideline: management of women with endometriosis. Hum Reprod. 2014;29:400–412. 24435778 10.1093/humrep/det457

[9] The Practice Committee of the American Society for Reproductive Medicine . Endometriosis and infertility: a committee opinion. Fertil Steril. 2012;98:591–598. 22704630 10.1016/j.fertnstert.2012.05.031

[10] Van Holsbeke C , Van Calster B , Guerriero S , et al. Endometriomas: their ultrasound characteristics. Ultrasound Obstet Gynecol. 2010;35:730–740. 20503240 10.1002/uog.7668

[11] Broekmans FJ , de Ziegler D , Howles CM , Gougeon A , Trew G , Olivennes F . The antral follicle count: practical recommendations for better standardization. Fertil Steril. 2010;94:1044–1051. 19589513 10.1016/j.fertnstert.2009.04.040

[12] Gardner DK , Lane M , Stevens J , Schlenker T , Schoolcraft WB . Blastocyst score affects implantation and pregnancy outcome: towards a single blastocyst transfer. Fertil Steril. 2000;73:1155–1158. 10856474 10.1016/s0015-0282(00)00518-5

[13] Benschop L , Farquhar C , van der Poel N , Heineman MJ . Interventions for women with endometrioma prior to assisted reproductive technology. Cochrane Database Syst Rev. 2010;11:CD00857. 10.1002/14651858.CD008571.pub2 PMC11608815 21069706

[14] Coccia ME , Rizzello F , Barone S , et al. Is there a critical endometrioma size associated with reduced ovarian responsiveness in assisted reproduction techniques? Reprod Biomed Online. 2014;29:259–266. 24947067 10.1016/j.rbmo.2014.04.019

[15] Tsoumpou I , Kyrgiou M , Gelbaya TA , Nardo LG . The effect of surgical treatment for endometrioma on in vitro fertilization outcomes: a systematic review and meta‐analysis. Fertil Steril. 2009;92:75–87. 18692796 10.1016/j.fertnstert.2008.05.049

[16] Almog B , Shehata F , Sheizaf B , Tan SL , Tulandi T . Effects of ovarian endometrioma on the number of oocytes retrieved for in vitro fertilization. Fertil Steril. 2011;95:525–527. 20378109 10.1016/j.fertnstert.2010.03.011

[17] Bussaca M , Riparini J , Somiglina E , et al. Postsurgical ovarian failure after laparoscopic excision of bilateral endometriosis. Am J Obstet Gynecol. 2006;195:421–425. 16681984 10.1016/j.ajog.2006.03.064

[18] Somigliana E , Arnoldi M , Benaglia L , Lemmell R , Nicolosi A , Ragno G . IVF–ICSI outcome in women operated on for bilateral endometriomas. Hum Reprod. 2008;23:1526–1530. 18441346 10.1093/humrep/den133

[19] Takashima A , Takeshita N , Otaka K , Kinoshita T . Effects of bipolar electrocoagulation versus suture after laparoscopic excision of ovarian endometrioma on the ovarian reserve and outcome of in vitro fertilization. J Obstet Gynaecol Res. 2013;39:1246–1252. 23803008 10.1111/jog.12056

[20] Li HW , Lee VC , Lau EY , Yeung WS , Ho PC , Ng EH . Ovarian response and cumulative live birth rate of women undergoing in‐vitro fertilisation who had discordant anti‐Müllerian hormone and antral follicle count measurements: a retrospective study. PLoS ONE. 2014;9:e108493. 25313856 10.1371/journal.pone.0108493 PMC4196774

[21] Sahmay S , Oncul M , Tuten A , Tok A , Acikgoz AS , Cepni I . Anti‐Müllerian hormone levels as a predictor of the pregnancy rate in women of advanced reproductive age. J Assist Reprod Genet. 2014;31:1469–1474. 25186502 10.1007/s10815-014-0324-y PMC4389927

[22] Broer SL , Mol B , Dólleman M . The role of anti‐Müllerian hormone assessment in assisted reproductive technology outcome. Curr Opin Obstet Gynecol. 2010;22:193–201. 20407372 10.1097/GCO.0b013e3283384911

[23] Al‐Inany HG , Youssef MA , Aboulghar M , et al. Gonadotrophin‐releasing hormone antagonists for assisted reproductive technology. Cochrane Database Syst Rev. 2011;11:CD001750. 10.1002/14651858.CD001750.pub3 21563131

